# Chemical Composition, Antioxidant and Antimicrobial Activity of Raspberry, Blackberry and Raspberry-Blackberry Hybrid Leaf Buds

**DOI:** 10.3390/molecules26020327

**Published:** 2021-01-10

**Authors:** Anna Krzepiłko, Roman Prażak, Agata Święciło

**Affiliations:** 1 Faculty of Food Sciences and Biotechnology, University of Life Sciences in Lublin, St. Skromna 8, 20-704 Lublin, Poland; anna.krzepilko@up.lublin.pl; 2 Institute of Plant Genetics, Breeding and Biotechnology, University of Life Sciences in Lublin, St. Akademicka 15, 20-950 Lublin, Poland; 3 Department of Environmental Microbiology, University of Life Sciences in Lublin, St. Leszczyńskiego 7, 20-069 Lublin, Poland

**Keywords:** leaf buds, *Rubus* genus, antioxidant, bioassay, antibacterial activity, mineral composition

## Abstract

In our investigation, the chemical composition and bioactive potential of leaf buds of raspberry, blackberry, and a raspberry-blackberry hybrid were determined. Antioxidant and antimicrobial properties were tested in water (W), ethanol-water (EW), and glycerol-water (GW) extracts from the buds. These plant organs contain relatively large amounts of minerals, especially Fe. The total antioxidant capacity (TAC) measured by the ABTS and DPPH methods ranged from 2.86 to 12.19 and 6.75 to 24.26 mmol per 100 g fresh weight (FW) of buds, respectively. TAC values were generally higher in the raspberry than in the case of blackberry and raspberry-blackberry hybrid extracts. The antioxidant properties of the extracts were strongly positively correlated with their content of total phenolic (TP). No such relationship was noted for ascorbic acid (AA), whose concentration in all extracts was at a similarly low level. Antioxidant properties determined in vitro were confirmed for the GW extract from raspberry leaf buds in biological test based on the growth parameters of Δ*sod1 Saccharomyces cerevisiae* mutant cells in hypertonic medium. The extracts also exhibited strong antibacterial properties against *Staphylococcus aureus* and *Enterococcus faecalis* and weaker against *Enterobacter aerogenes*. The studied leaf buds could be therefore an unconventional source of minerals, natural antioxidants and antibacterial compounds with potential applications in the food, pharmaceutical, and cosmetics industries.

## 1. Introduction

Leaf and flower buds of many plant species, due to their high content of biologically active substances, are used in pharmacology, cosmetics, and food production. These plant organs have meristematic tissues containing concentrated active substances, often more diverse in composition and quantity than fully developed organs [1].

Leaf buds of berry shrubs are a material of relatively low popularity and a narrow range of applications. In contrast, their edible fruits called berries have long been known and appreciated for their nutritional value. They are a valuable component of the diet, rich in bioactive substances and minerals. *Rubus* fruits, such as raspberries, blackberries, and hybrid berries, have high content of vitamins (C, A, E, B1, B2, B3, B6, and K), organic acids (citric, malic), phenolic acids (derivatives of cinnamic and benzoic acids), polyphenols, cyanidin, aromatic compounds, pectins, minerals, sugars, and dietary fibre [2,3]. In addition, they exhibit the health-promoting properties through anti-cancer, anti-atherosclerotic, anti-inflammatory, anti-diabetic, and anti-allergic effects, and also reduce body weight [4]. For this reason, raw raspberry and blackberry and their food preserves are highly valued by nutritionists and herbalists. However, fresh fruits of berry shrubs are available seasonally, while food and herbal products made from them lose many of the valuable components that determine their health-promoting properties during processing. As an alternative, these bioactive substances could be obtained from other organs of these plants, such as young shoots, leaf buds or leaves, which are available throughout the growing season.

In folk medicine, blackberry leaves and young shoots were used to relieve pain and treat hemorrhoids, as well as for their anti-diarrheal effects and their anti-inflammatory effects in infectious diseases of the oral and pharyngeal mucosa. In addition, extract from the leaves was used as a relaxant, especially for uterine muscles, and for its beneficial effects during pregnancy [5]. Scientific research has confirmed that the leaves of these shrubs, like their fruit contain a variety of compounds with health-promoting and antioxidant properties [6,7,8]. However, knowledge of the composition and health-promoting effects of bioactive substances contained in the buds of species of the genus *Rubus* is not as widespread as knowledge of the properties of their leaves and fruits. Another argument in favour of research on this subject is reports suggesting that young plant organs usually have higher content of various bioactive compounds than older ones, which suggests that the leaf buds of berry bushes, as juvenile organs, could be particularly good reservoirs of them [1].

The aim of the study was to analyze of raspberry, blackberry, and raspberry-blackberry leaf buds, primarily in order to determine their chemical composition and biological properties. The content of minerals, i.e., potassium (K), calcium (Ca), magnesium (Mg), iron (Fe), manganese (Mn), zinc (Zn), and copper (Cu) was determined in the buds, and the antioxidant and antimicrobial capacity of water (W), ethanol-water (EW) and glycerol-water (GW) extracts from these buds was tested. Antioxidant properties were determined using classic chemical methods (determination of the content of total phenolic (TP) and ascorbic acid (AA)), biochemical methods (determination of the capacity to scavenge artificial ABTS^•+^ and DPPH^•^ radicals) and an innovative bioassay exploiting the phenomenon of restoration of growth to Δ*sod1* mutant *S. cerevisiae* yeast cells in a hypertonic medium [9,10]. The advantage of the biological method is that it not only confirms the antioxidant properties of the components of the samples in vivo, but also provides additional information about their bioavailability for eukaryotic cells and their non-specific cellular effects. Moreover, the antimicrobial potential of the extracts against different opportunistic bacteria for humans was tested.

## 2. Results

### 2.1. Mineral Content in Leaf Buds

The concentration of minerals, i.e., Ca, K, and Mg (macroelements) and Fe, Cu, Mn, and Zn (microelements) varied in the raspberry, blackberry and raspberry-blackberry hybrid leaf buds. The highest content of K was noted in the buds of ‘Navaho’ blackberry, and the highest contents of Ca and Fe in the buds of ‘Glen Fyne’ raspberry (Table 1). Raspberry-blackberry hybrid buds had the highest levels of Mg and Mn, raspberry buds of the ‘Octavia’ cultivar had the highest content of Zn, and the highest content of Cu was found in the buds of wild blackberry (Table 1).

The largest differences in K content (238.61 mg 100 g^−1^) in the buds were found for wild blackberry and its cultivated form ‘Navaho’. In terms of Ca content, the ‘Glen Fyne’ and ‘Navaho’ varieties differed the most (68.92 mg 100 g^−1^). The greatest differences in Mg content were found for the raspberry-blackberry and wild blackberry (34.88 mg 100 g^−1^); in Fe content for ‘Glen Fyne’ and ‘Navaho’ (12.25 mg 100 g^−1^); and in Zn content for the ‘Octavia’ and the raspberry-blackberry hybrid (1.32 mg 100 g^−1^).

### 2.2. TP and AA Contents

**Figure 1:**
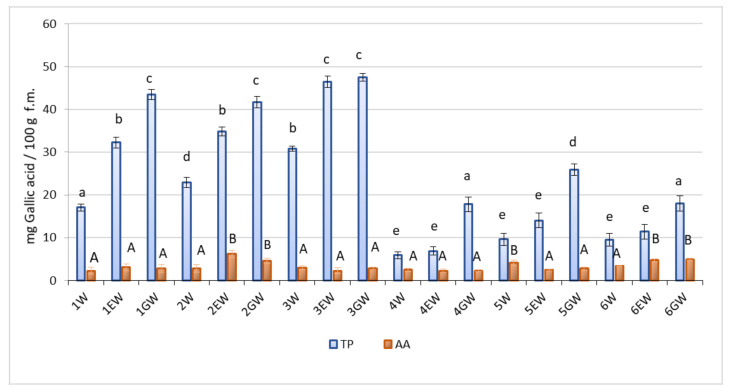
TP and AA content in buds extracts. Forms: 1—‘Cascade Delight’, 2—‘Glen Fyne’, 3—‘Octavia’, 4—‘Navaho’, 5—wild blackberry, 6—raspberry-blackberry hybrid. Extracts: W—water, EW—ethanol-water, GW—glycerol-water. Error bars represents ±SD. Different lowercase letters (for TP) or uppercase letters (for AA) indicate significant differences at α = 0.05.

A modified F-C assay was used to determine the content of TP and AA [11]. Owing to this approach, the same method could be used to determine both groups of compounds which are usually present in plant raw material. The specificity of the F-C method was improved by calculating of a corrected TP value based on the AA reducing activity present in the extract (Figure 1).

TP content in the bud extracts of the *Rubus* species and cultivars fell within a fairly wide range, from 5.89 mg 100 g^−1^ to 47.45 mg 100 g^−1^ (Figure 1). This parameter also varied depending on the plant genotype and solvent used. TP content was higher in the raspberry cultivars, and among them, the highest in the ‘Octavia’ (30.85–47.45 mg 100 g^−1^, depending on the solvent used). In blackberry buds, the TP content ranged from 5.89 to 25.88 mg 100 g^−1^ and was highest in the GW extract from buds of wild variety. The raspberry-blackberry buds contained comparable amounts of TP as the blackberry buds, but significantly smaller amounts of these phytochemicals than raspberry buds. For all genotypes, the highest TP content was noted in GW extracts, and the lowest in W extracts (Figure 1).

The content of AA in the extracts from the leaf buds of the raspberry, blackberry, and raspberry-blackberry hybrid varieties ranged from 2.26 mg 100 g^−1^ to 6.19 mg 100 g^−1^ FW. Higher AA content was found in the EW and GW extracts from the buds of ‘Glen Fyne’ raspberry and tayberry and in the W extracts from wild blackberry. In the remaining extracts, irrespective of the type of solvent used, the values were low and did not differ statistically (Figure 1).

### 2.3. Total Antioxidant Capacity

**Figure 2:**
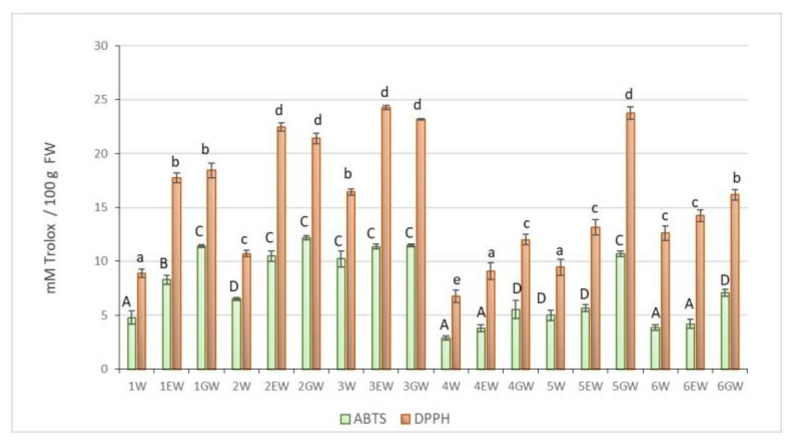
TAC in buds extracts determined by ABTS and DPPH methods. Forms: 1—‘Cascade Delight’, 2—‘Glen Fyne’, 3—‘Octavia’, 4—‘Navaho’, 5—wild blackberry, 6—raspberry-blackberry hybrid. Extracts: W—water, EW—ethanol-water, GW—glycerol-water. Error bars represents ±SD. Different lowercase letters (for DPPH method) or uppercase letters (for ABTS method) indicate significant differences at α = 0.05.

Higher values of the antioxidant capacity were obtained using DPPH than ABTS method. TAC determined by the DPPH method varied between genotypes, with the relatively high values obtained in the EW and GW raspberry extracts of the ‘Octavia’ (24.26 mmol 100 g^−1^ and 23.17 mmol 100 g^−1^, respectively) and ‘Glen Fyne’ (22.46 mmol 100 g^−1^ and 21.41 mmol 100 g^−1^, respectively) cultivars and in the GW of wild blackberry extract (23.76 mmol 100 g^−1^) (Figure 2).

Antiradical activity determined by the ABTS method was generally higher in the extracts of raspberry cultivars than in extracts of blackberry and raspberry-blackberry hybrid (Figure 2). In the case of raspberry extracts, similar, high antioxidant capacity was noted for the EW and GW extracts. In the case of the blackberries and the raspberry-blackberry hybrid, the highest TAC measured by the ABTS method was found in the GW extracts.

There was a high positive correlation between TP content and TAC determined by the ABTS method (r = 0.94) and the DPPH method (r = 0.84) in the extracts of the leaf buds. The ABTS and DPPH methods of determining TAC were also highly correlated (r = 0.90). Content of AA was not correlated with TAC measured by the ABTS methods, but a weak positive correlation was established between AA and TAC measured by the DPPH method (r = 0.28) (Table 2).

The solvent used for extraction affected the content of antioxidants in the extracts. The highest content of antioxidants reacting specifically with ABTS radical cation was found in GW extracts, and antioxidants reacting with DPPH radical in EW and GW extracts. On the other hand, water extracts were relatively poor in both types of substance (Figure 2).

Correlations between the TP content and TAC (determined by ABTS and DPPH methods) in different types of extracts (W, EW, and GW) prepared from the raspberry, blackberry and hybrid leave buds were assessed as well. The highest positive correlations between TP content and TAC (measured using ABTS and DPPH tests) and between both antioxidant tests (ABTS and DPPH) were found in the EW extract. The lowest positive correlations between TP and TAC were established in GW extracts and between ABTS and DPPH tests in W extract (Table 3).

### 2.4. Antioxidant Yeast Test Based on Growth ∆sod1 Mutant in Hypertonic Medium

**Figure 3:**
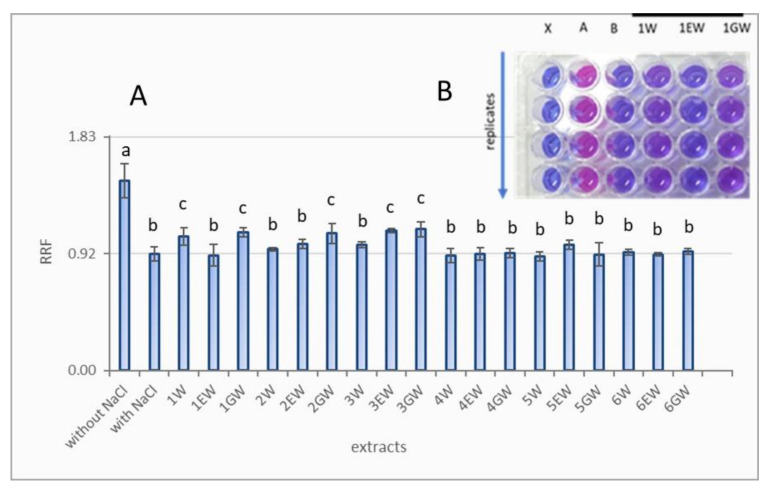
(**A**) Recovery of growth of Δ*sod1 S. cerevisiae* mutant in hypertonic medium in the presence of raspberry, blackberry and tayberry leaf bud extracts. Forms: 1—‘Cascade Delight’, 2—‘Glen Fyne’, 3—‘Octavia’, 4—‘Navaho’, 5—wild blackberry, 6—raspberry-blackberry hybrid. Extracts: W—water, EW—ethanol-water, GW—glycerol-water. Error bars represents ±SD. Different lowercase letters indicate significant differences at α = 0.05. (**B**) Colorimetric reaction of resazurin dye showing the effects of 1W, 1EW, 1GW extracts on growth intensity of Δ*sod1 S. cerevisiae* culture. Column X: PBS without a yeast suspension, column A: Δ*sod1* culture on standard YPD medium (positive control), column B: Δ*sod1* culture on hypertonic medium (negative control), columns 1W to 1GW: Δ*sod1* culture on hypertonic medium with the addition of an appropriate extract.

The antioxidant properties of extracts from raspberry, blackberry and tayberry leaf buds were tested using a bioassay exploiting the phenomenon of restoration of growth to Δ*sod1 S. cerevisiae* mutant cells in a hypertonic environment. The capacity to restore the growth of these cells in the presence of extracts was monitored spectrophotometrically after adding the dye resazurin to three-day old yeast cultures. The level of reduction of resazurin reflected the density of these yeast cultures (Figure 3).

The strongest antioxidant properties were observed in the GW extracts from the leaf buds of all raspberry cultivars, the EW extract from the ‘Octavia’ raspberry cultivar, and the W extracts from the ‘Cascade Delight’ raspberry cultivar. The respective RRFs for these extracts were 1.08 ± 0.04 (GW ‘Cascade Delight’), 1.08 ± 0.08 (GW ‘Glen Fyne’), 1.11 ± 0.06 (GW ‘Octavia’), 1.10 ± 0.02 (EW ‘Octavia’), and 1.05 ± 0.07 (W ‘Cascade Delight’), and were significantly higher than the RRF of the yeast culture without the addition of extract (Figure 3A). The remaining extracts were not effective in this assay. The degree of stimulation of the growth of yeast by the effective extracts was small compared to a culture growing in optimal conditions, i.e., in a medium without an inhibitory action of 0.8M NaCl.

### 2.5. Antibacterial Activity of Leaf Bud Extracts

The antibacterial activity of raspberry, blackberry, and raspberry-blackberry hybrid bud extracts against selected opportunistic bacteria for humans was determined by the disc-diffusion method. No growth inhibition zones were found for *Escherichia coli*, *Serratia marcescens*, *Salmonella enterica*. In the case of *E. aerogenes,* only the EW extract had a weak effect (inhibition zones 1.3–3.5 mm; Table 4).

The extracts were found to affect the growth of *S. aureus* and *E. faecalis*. High antimicrobial activity against *E. faecalis* was found for GW extracts from the buds of all forms (inhibition zones 7.5–14.5 mm). A similar but weaker relationship was noted in the case of *S. aureus* (inhibition zones 4.0–8.0 mm; Table 4).

There was a high, positive, statistically significant correlation at *p* < 0.05 between the amount of TP and antioxidant substances (determined both by the ABTS and DPPH methods) applied to the disc with the extract and the size of the growth inhibition zones for *S. aureus* and *E. faecalis*. No correlations were found for *E. aerogenes*.

## 3. Discussion

Minerals—macro- and microelements—are necessary for maintaining human health, and their sources in the diet are foods of plant or animal origin and water. The results indicate that K is the most abundant of the studied elements in the leaf buds of the *Rubus* genus. In addition, we found large amounts of Ca, Mg, and Fe in all tested samples. There were small, often statistically insignificant differences in the content of minerals between the buds of the raspberry, blackberry, and raspberry-blackberry genotypes, which can be explained by the fact that the fertilization level, location, and genus were the same in all cases (Table 1). These factors largely determine the quantity of phytochemicals in plant material [12,13]. There were no statistically significant differences between raspberry cultivars in the content of K, Mg, or Cu in the buds. Raspberry-blackberry hybrid buds did not differ significantly from the raspberry cultivars in terms of K, Ca and Fe content, but they contained statistically significantly more Mg, Mn, and Cu and less Zn than the raspberries. Compared to ‘Navaho’ blackberry, the raspberry-blackberry hybrid did not differ statistically in K or Cu content, but contained significantly more Ca, Mg, Fe, and Mn.

To our knowledge, there are no literature reports regarding the content of the elements tested in the raspberry and blackberry buds, so they can only be compared to the leaves or fruit of these plants. In raspberry leaves studied by Horuz et al. [14] and Karaklajić-Stajić et al. [15], Fe content ranged from 7.8 mg 100 g^−1^ to 12.3 mg 100 g^−1^ and was much lower than the values obtained in our study for raspberry buds (15.90–24.02 mg 100 g^−1^). Kessel [16] found even smaller Fe content in raspberry leaves ranging from 0.5 to 2 mg 100 g^−1^, while Dresler et al. [17] reported that the average Fe content in raspberry leaves was similar to the content of this element in buds and amounted to 19.1 mg 100 g^−1^.

However, the content of other elements in the buds was much lower than in raspberry leaves. Potassium content in raspberry leaves (660–1010 mg 100 g^−1^) was about one and a half times as high as in buds [14]. According to this research, raspberry leaves contain more than ten times as much Ca (1370–2110 mg 100 g^−1^), Mn (6.3–7.7 mg 100 g^−1^), and Zn (4.5–7.3 mg 100 g^−1^). The magnesium content in the raspberry leaves (330–520 mg 100 g^−1^) was over three times higher than that in the buds [14]. Similarly, copper content in leaves (1.082–2.273 mg 100 g^−1^) also significantly exceeds the amount we found in the buds. Other authors also have reported much higher content of K, Ca, Mg, Mn, Zn, and Cu in raspberry leaves than we found in the buds [17].

The content of mineral elements determined in dry matter of raspberry fruits, i.e., K (118–121 mg 100 g^−1^), Ca (2.2–2.3 mg 100 g^−1^), Mg (6.3–6.7 mg 100 g^−1^), Fe (0.14–0.2 mg 100 g^−1^), Zn (0.08–0.18 g 100 g^−1^), and Mn (0.28–0.48 mg 100 g^−1^), was lower than the content determined in our research in leaf buds [18]. Only the content of Cu in the fruit was comparable to the content of this element in the leaf buds.

The opposite tendency was observed when the mineral composition of blackberry fruit and leaf buds of this shrub were compared. Blackberry fruit contains more K, Mg, Mn and Zu than the studied leave buds but similar amounts of Ca and Zn. However, they contain much less iron than the leaf buds [19]. Blackberry leaves contain similar amounts of macronutrients (such as K, Ca, Mg) as buds, but about five times more manganese and twice as much copper. However, as in the case of fruit, they contain much less iron than the leaf buds [19,20].

The examples cited and the results of the present study indicate that variation in the mineral composition of plant material is substantial and depends, inter alia, on the plant genotype, its organ, and its physiological maturity, as well as on climate and soil conditions, agrotechnical procedures, and the mobility of a given element in the plant [14,15,16,17,18,19,20]. The leaf buds of raspberry are generally richer in minerals than the fruits, but poorer than the leaves (except for Fe, whose content is higher in the leaf buds of this plant than in the leaves). Blackberry leaf buds, on the other hand, contain similar amounts of K and Ca to the leaves and fruits of this plant, slightly less Mg but are richer in Fe and Zn. The average iron content in the analyzed leaf buds was comparable to its average content in leafy green vegetables considered to be very good sources of this element, such as lettuce, spinach, and kale, and it was much higher than in cabbage, parsley (leaves and root), leek, carrot, celery, tomato, and cucumber [21,22].

Another challenge was to comprehensively investigate the antioxidant properties of the phytocompounds in raspberry, blackberry, and raspberry-blackberry buds. Various methods—chemical, biochemical and biological—were used for this purpose. TP content ranged from 5.89 mg∙100 g^−1^ to 47.45 mg∙100 g^−1^ FW and depended on the solvent type and genotype. The highest content of phenolic compounds was found in the GW and EW extracts from the raspberry leaf buds. The blackberry and raspberry-blackberry leaf buds had markedly lower content of this type of compound. The reverse tendency was noted in previous research on extracts from fruits of these plant species [9,23]. In these cases, the phenolic content of fruits of blackberry and *Rubus* hybrid was much higher than in the fruits of the raspberry cultivars. Thus, the genetic factor determines the unique chemical composition of secondary metabolites of various plant functional organs [1].

In our research on the same genotypes, the TP content of aqueous extracts from the fruits of blackberry (cultivated and wild) and the raspberry-blackberry hybrid was more than ten times as high as in the leaf buds of these shrubs studied in the present study [9]. In the case of raspberry, such large disproportions between leaf buds and fruits were not observed. The aqueous extracts from these fruits contained similar amounts of TP as the leaf buds of these shrubs. In the case of fruit, anthocyanins accounted for about half of the total content of phenolic compounds, while their share was negligible in the case of the buds [9]. Only trace amounts of anthocyanins were noted in the studied buds of these shrubs, and the differences between genotypes were small. For this reason, these results were not included in this paper. The data suggest that the qualitative composition of phenolic compounds in the leaf buds of shrubs of the genus *Rubus* and their fruits is varied.

In our research, TAC in the raspberry, blackberry, and raspberry-blackberry buds reached values in the range of 2.86–12.19 mmol∙100 g^−1^ when measured by the ABTS method, and 6.75–24.26 mmol∙100 g^−1^ by the DPPH method. Other researchers obtained similar TAC values for the fruit of these shrubs. Sariburun et al. [24] found that TAC in aqueous extracts of raspberry fruit was 6.4–8.3 mmol∙100 g^−1^ measured by the ABTS method and 6.4–8.9 mmol∙100 g^−1^ by the DPPH method. In the case of blackberry fruit, TAC in the ABTS test was 7.4–9.3 mmol∙100 g^−1^, while in the DPPH test it was 1.9–2.3 mmol∙100 g^−1^.

Raspberry bud extracts had higher TP content and TAC measured by the ABTS and DPPH methods than blackberry extracts (Figure 1 and Figure 2). The wild blackberry bud extracts, except for the GW extracts, studied by the ABTS and DPPH methods, did not differ from the extracts from cultivated blackberry or raspberry-blackberry hybrid buds. Milivojevic et al. [25] found different relationships in fruits of these berry shrubs: wild blackberry fruits had the highest TP content and the highest antioxidant potential determined by the ABTS method. These parameters decreased in the fruits in the following order: blackberry fruit, cultivated raspberry fruit, and wild raspberry fruit. These data support the opinion of other researchers that the content of phenolic compounds in *Rubus* organs can be varied due to the genotype of the plant and various production factors, including environmental conditions [26].

To establish a quantitative relationship between TAC and TP content, a correlation analysis was performed (Table 2). A high positive correlation was obtained, which indicates that the antioxidant capacity of the studied buds extracts was largely due to the presence of phenolics compounds. This relationship was found in all extracts, but the highest correlations between these parameters were found in EW and the lowest in GW extracts (Table 3). Ethanol-water solutions are known for their high efficiency in extracting phenolic compounds from raw plant material, which may explain this phenomenon [27,28]. Other authors have also confirmed the relationship between TP content and TAC of plant extracts [19,25,29,30]. However, it is believed that the antioxidant activity of plant extracts is not determined by the presence of polyphenols alone, so determinations by other constituents are fully justified.

In our study, in addition to TP content in the buds extracts, we also examined the AA content. However, it was low in comparison to its content in the fruits of the same *Rubus* species and varieties [9]. Moreover, the content of AA was not found to be correlated with the TAC of the extracts measured by the ABTS method, and only a weak correlation was established with TAC measured by the DPPH method (Table 3). The literature data confirm that, like in buds also in the fruits of these shrubs, AA is not an influential contributor to their antioxidant capacity [29,31,32].

Strong antiradical activity of plant extracts, expressed in vitro, is not always adequate for their antioxidant capacity following ingestion, i.e., in vivo. For this reason, we examined raspberry, blackberry and raspberry-blackberry leave bud extracts using yeast growth assay. In this case the antioxidant mechanism involves the ability of bioactive compounds to penetrate cells and affect cellular metabolism by reacting specifically with reactive oxygen species (ROS) or products of their activity. The effects of antioxidants were monitored using various biochemical or physiological indicators. In our study, the antioxidant property of extract constituents was expressed as their capacity for the stimulation of Δ*sod1 S. cerevisiae* cells growth. This phenomenon has been observed after application to the hypertonic medium mainly GW extracts from the leaf buds of raspberry cultivars (Figure 3A,B). Extracts of blackberry and raspberry-blackberry were not effective in this test.

Biological activity of GW extracts from raspberry most likely results from their high content of TP (Figure 1), which include compounds with a documented positive effect in this bioassay, such as gallic acid and salicylate [9]. Raspberry leaves are richer in salicylate than blackberry leaves, which may suggest that the same relationships occur in young leaves and leaf buds [33]. The role of AA, unlike our previous research seems to be minor, because its content in all the extracts was low, with little variation [9,10]. It is possible that the extracts also contained considerable amounts of other bioactive compounds, such as reduced glutathione or sulphur-containing amino acids, which also had a positive result in this test [9,11]. Despite the fact that natural antioxidants in plant extracts are present in small quantities and varying proportions, they can induce strong biological effects owing to their additive or synergistic effect. Such effects have been observed in the case of many pure natural or synthetic antioxidants, plant extracts and plants foods in biochemical and biological tests [34,35,36].

In addition to the antioxidant properties of the leaf bud extracts, their antibacterial properties were analyzed as well. Representatives of the *Enterobacteriaceae* family were selected for the antimicrobial test. Most of them are harmless species causing no disease symptoms, commonly found in water and soil, as well as inhabiting the human digestive tract and skin surface [37]. A large group of bacteria belonging to this family comprises opportunistic microorganisms, which pose a threat to the weak, the elderly and the sick [37].

Among the group of six selected bacteria, two species (*E. faecalis* and *S. aureus*) proved susceptible to all the extracts, with the GW extracts found to be most effective. *E. aerogenes* exhibited relatively low susceptibility only to EW extracts from the berry leaf buds. Three other species, *S. enterica*, *S. marcescens*, and *E. coli*, proved to be completely resistant to the active substances in the extracts.

*S. aureus*, which was susceptible to the active substances in the extracts, is recognized as one of the most important agents of nosocomial infections [38]. Another sensitive bacterium, *E. faecalis* is commonly found in the digestive tract of humans and animals as an element of the commensal bacteria. It is also a clinically important species because it causes severe enterococcal infections, including infective endocarditis [39]. Drug resistance is becoming an increasing challenge for modern medicine, and the risk of morbidity and mortality associated with resistance of pathogenic microorganisms to most drugs is increasing. The data obtained indicate potential antibacterial uses of bud extracts, e.g., to prevent *S. aureus* and *E. faecalis* infections.

The susceptibility of *E. faecalis* and *S. aureus* to the components of the extracts was positively correlated at *p* < 0.05 with their content of TP and of antioxidant potential expressed as TAC (Table 4). No such correlations were found for *E. aerogenes*.

Phenolic compounds are also known to show antimicrobial activity, with their effectiveness depending on their structure and the type of microorganism [40]. Due to the large variation in the structure of these compounds, their antimicrobial activity is mediated through a variety of mechanisms. Phenolic compounds have the ability to interact with the cytoplasmic membrane, cell wall, nucleic acids, and respiratory chain enzymes, thereby altering or inhibiting their function [41,42,43]. Phenolic acids also denature enzymes or bind vitamins, minerals or carbohydrates, so that these compounds become inaccessible to microorganisms [44].

One of the key factors determining the biological activity of substances in extracts is their concentration, which depends on how effectively they are extracted from plant material. Here the type of solvent used plays an important role. Commonly used solvents for the extraction of bioactive compounds from plants include water, ethanol, methanol, chloroform, dichloromethanol, ether, and acetone, alone or in mixtures [45]. During preparation of bioactive substances by extraction, residues of toxic or harmful solvents may remain. In the present study, the solvents used to prepare the extracts were water, ethyl alcohol and glycerol, which are safe for the human body and the environment according to the standards set by the Environmental Protection Agency, USA. They are non-toxic to humans and microorganisms used in these studies. The procedures we used to obtain the extracts have been recommended by other authors, including the ratio of the volume of solvent to the weight of the sample (10:1 *v*/*w*), homogenization of plant tissues in the solvent, and centrifugation to obtain the extract [46,47]. Taking into account the antioxidant and antimicrobial abilities of the examined extracts, the efficiency of the extractants can be ranked in the following order: 40% (*v*/*v*) glycerol-water solution, 40% (*v*/*v*) ethanol-water solution, and pure water.

## 4. Materials and Methods

### 4.1. Plant Material

Leaf buds of raspberry *Rubus idaeus* L.-‘Cascade Delight’, ‘Glen Fyne’ and ‘Octavia’ varieties; thornless blackberry *Rubus fruticosus* L.-‘Navaho’ variety; blackberry *Rubus fruticosus* L.-native population; and *Rubus idaeus* L. × *Rubus fruticosus* L. hybrid were collected at the beginning of March during the bud break stage. According to literature data, the content of bioactive compounds is highest in the case of *Rubus* plants during this stage of phenological development [1]. Plants were grown in the experimental field of the Felin Experimental Station of the University of Life Sciences in Lublin (51°13′34.1″ N 22°38′15.2″ E), on soil formed of silt of loess origin, classified as good wheat complex. During the growing season, basic agrotechnical treatments were performed and NPK fertilization was applied (N—120 kg ha^−1^, P—60 kg ha^−1^, K—20 kg ha^−1^).

The dry weight of leaf buds was determined by the drying method at 70 °C until constant weight was obtained. The dry weight of buds varied for individual genotypes from 9.1 to 9.6%

### 4.2. Microorganisms

Reference strains of bacteria were obtained from the Polish Collection of Microorganisms (Wroclaw, Poland): *Enterobacter aerogenes* PCM 1836, *Escherichia coli* PCM 2337, *Serratia marcescens* PCM 549, *Salmonella enterica* serovar Enteritidis PCM 930, *Staphylococcus aureus* PCM 2267 and *Enterococcus faecalis* PCM 2786. The strains were stored in a deep freeze. Prior to the experiment, they were transferred onto nutrient agar slants (Sigma-Aldrich, Darmstadt, Germany) and incubated under optimal conditions. To determine the antioxidant properties of bud extracts using a biological test, a mutant Δ*sod1* strain of the yeast *Saccharomyces cerevisiae* with the genotype MAT α leu1 arg4 *sod1*::natMX was used [11]. Yeast was grown in standard liquid YPD medium (1% yeast extract, 1% yeast bacto-peptone, and 2% glucose) without any additives or with the addition of 0.8 M NaCl to get a hypertonic environment.

### 4.3. Determination of Mineral Composition

The buds were crushed, dried and ground, and the content of K, Ca, Mg, Mn, Fe, Zn and Cu was determined (after wet mineralization in extra pure HNO_3_) by atomic adsorption spectroscopy according to PN-EN ISO 6869:2002 [48]. Elements were analyzed in three laboratory samples prepared from buds of the test plants. The results were expressed in mg per 100 g dry weight (DW) of buds.

### 4.4. Extract Preparation

Water (W), ethanol-water (EW, 40% *v*/*v*) and glycerol-water (GW; 40% *v*/*v*) extracts from the buds were made using single-stage maceration with mixing. The extract was prepared by mixing previously washed and air-dried buds with the extraction solution in a 1:10 weight ratio. The plant material was ground with a mechanical homogenizer for 5 min while the samples were cooled in an ice bath. The homogenate was mixed for 24 h on a laboratory shaker at 60 rpm and 22 °C, and then centrifuged for 5 min at 5000 rpm. The supernatant was frozen and used for further analysis.

### 4.5. Determination of Total Antioxidant Capacity (TAC)

The TAC was determined in the extracts by the ABTS and DPPH method. ABTS (2,2’-azobis (3-ethylbenzothiazoline-6-sulfonate) forms the ABTS^•+^ radical cation, which is reduced by the antioxidants present in the extract, leading to the loss of the blue-green colour of the solution. The change in absorbance, which depends on the antioxidant content of the sample, was measured spectrophotometrically at 414 nm, 30 min after the reagents were mixed [49].

During the reaction of DPPH (2,2-diphenyl-1-picrylhydrazil) with an antioxidant, the stable DPPH^•^ radical takes electrons from the antioxidant, leading to the loss of its violet colour [50]. The decrease in absorbance relative to the control was measured at 517 nm [51].

In both methods, with ABTS and DPPH, the TAC was expressed as trolox equivalent (TE) per 100 g FW of buds [49,50].

### 4.6. Determination of TP and AA Content

The content of TP and the sum of AA and its oxidized form dehydroascorbic acid (DHA) in the extracts was determined by spectrophotometry using the Folin-Ciocalteu (F-C) reagent. Due to the very small amount of DHA in the fresh material, the abbreviation AA will hereafter refer to AA + DHA. According to the strategy proposed by Sanchez-Rangel et al. [11], both types of phytocompounds can be assayed using the same reagent, because they react with it in different, specific pH ranges. Under acidic conditions (pH = 3), AA rapidly reacts with phosphotungstate (the main component of the F-C reagent), resulting in a blue colour immediately after the plant extract is mixed with the reagent. The AA content is determined by measuring the absorbance of this solution at 765 nm. Then sodium carbonate is added to the mixture according to the standard protocol for the F-C assay to raise the pH of the solution to about 10, which allows phenolic compounds to react with the components of the F-C reagent [52]. The absorbance is read after the solution has been incubated for 1 h in the dark at the same wavelength as previously. The TP results obtained using this procedure were expressed as gallic acid equivalent (GAE) per 100 g FW of buds. The TP result was corrected by subtracting the value for the AA reducing activity obtained in the same assay. For this purpose, AA reducing activity was expressed in the same units as TP, i.e., GAE, according to the formula given by Isabelle et al. [53]: AA reducing activity = AA content (mg mL^−1^) × 0.872.

### 4.7. Antioxidant Yeast Test

The theoretical basis for this assay was developed by Żyracka et al. [54] and Koziol et al. [55] and its suitability for assessment of the antioxidant properties of raw plant extracts has been confirmed by Święciło et al. [9] and by Święciło and Rybczyńska-Tkaczyk [10].

The assay involves monitoring the growth parameters of Δ*sod1* mutant *S. cerevisiae* cells under severe osmotic stress (OS) induced by NaCl in the presence of the extracts. Severe OS is believed to generate symptoms of oxidative stress in yeast cells [55]. Besides the exogenous oxidative stress induced by the presence of NaCl, Δ*sod1* mutant yeasts are also continually exposed to endogenous oxidative stress caused by the lack of activity of cytoplasmic superoxide dismutase (Cu, Zn SOD), an enzyme that scavenges superoxide radical from this cellular compartment. This is probably why they are more susceptible than wild-type yeast cells to pro-oxidative factors and various environmental stressors, including OS [55,56,57]. One manifestation of this hypersensitivity is a slowing down or complete inhibition of the growth of these cells. Growth defects in Δ*sod1* yeast induced by severe OS can be eliminated by adding certain pure antioxidants to the growth media, mainly thiols and a few of the phenolics [9,11,54]. Restoration of growth to these cells can thus be used to detect these antioxidants in complex environmental or food samples. Furthermore, as the extent to which the growth of the yeast is restored (fully or partially) is concentration-dependent, the assay can also be used to estimate their content in samples.

Δ*sod1* mutant cells were grown in liquid YPD medium without any additives (positive control) or with 0.8 M NaCl (negative control) and with 0.8 M NaCl and leaf bud extracts in a volume of 5% *v*/*v*. Our previous research showed that this is the optimum volume of extracts, as they exert a biological effect without significantly altering the osmotic conditions of the medium. The yeast cultures were run for 3 days at 28 °C on a rotary shaker. Then the density of the culture was determined using a resazurin reduction assay [58].

Resazurin (7-hydroxy-3*H*-phenoxazin-3-one 10-oxide) dye has been broadly used as a cell viability indicator in many cytotoxicity and cell growth proliferation assays as it is a rapid, non-radioactive, non-toxic, low cost and highly sensitive method [59]. Resazurin enters the cell in the oxidized form (blue) and is converted to the reduced form, resorufin (pink). The reduced and oxidized forms of resazurin can be separately measured by a spectrophotometer and used to determine the reduction capability of cells, which reflects the status of mitochondrial function, cell viability and shows the growth inhibition level of cells.

Briefly, three-day yeast cultures were centrifuged and suspended in phosphate-buffered saline (PBS). Then 180 μL of yeast suspension was introduced into each well of a 90-well microplate and 20 μL of a 60 μM resazurin solution in PBS buffer was added. Immediately after this addition and after 1 h incubation of the plates in the dark, their absorbance was measured at two wavelengths 570 nm and 600 nm and calculating resazurin reduction factor (RRF).

### 4.8. Determination of the Antimicrobial Activity of the Extracts

The biostatic effect of the extracts on selected strains of opportunistic bacteria for humans was determined by the disc-diffusion method in accordance with EUCAST methodology [60]. A bacterial suspension with a density of 0.5 McFarland in 0.9% NaCl was applied to Mueller-Hinton agar. Filter paper discs were placed on the surface and 10 µL of bud extract was applied to them. Plates were incubated at 37 °C for 24 h. Growth inhibition zones were measured. A kanamycin disc (30 µg/disc) was used as a positive control. Blank disc impregnated with the respective solvent, were used as negative control but none of the solvents (10 µL/disc) showed antibacterial activity.

### 4.9. Statistical Analysis

All tests were performed in a minimum of three replicates. The results were subjected to analysis of variance. The average TP and AA contents and TAC obtained for the W, EW, GW extracts are shown in Figure 1 and Figure 2. The significance of differences was determined using the Tukey test. Results were considered statistically significant at α = 0.05. Values marked with the same letters indicate a lack of statistically significant differences between groups. The correlation between variables was examined using the Pearson (r) linear correlation coefficient.

## 5. Conclusions

Leaf buds of raspberry, blackberry, and raspberry-blackberry contain relatively high levels of minerals (K, Ca, Mg, Fe, Cu, Mn, and Zn) that are essential in the human diet as well as high concentrations of bioactive substances with antioxidant and antimicrobial properties. Due to the high content of Fe and Zn, deficient elements in the human diet the buds of these *Rubus* shrubs, can be used for the production of dietary supplements, especially for vegetarians. Although the non-haem iron contained in foods of plant origin is not as well absorbed (about 1–15%) as the haem iron in meat (≈15–40%), both of these forms are absorbed in the human body [61]. Thus, *Rubus* leaf buds can enrich the human diet, including vegetarian diets, with these elements. Diversification of vegetarian diets with products based on buds will provide also wider range of other minerals and may improve health quality of this food.

Our study showed that the leaf buds of raspberry, blackberry, and raspberry-blackberry hybrid contain a number of bioactive substances that are easily extracted using widely available, inexpensive, non-toxic solvents and exhibit anti-radical abilities in vitro. Raspberry leaf buds are particularly rich in these substances. Furthermore, antioxidants from raspberry leaf buds are able to penetrate eukaryotic cells and affect cellular metabolism in such a way as to eliminate the adverse effects of severe oxidative stress. A perceptible effect of their intracellular activity is the restoration of the cell cycle of *S. cerevisiae* yeasts. The beneficial effect of raspberry extracts on the physiological processes of this model eukaryotic organism, suggests that they might have similar effects in higher organisms such as animals and humans. Thus, they could find application in the prevention or treatment of disease conditions caused by severe oxidative stress.

In gemmotherapy, based on the healing properties of meristematic tissues, biological effects result from the synergistic or additive action of bioactive compounds and other plant components [1]. It cannot be ruled out that this type of effect plays a major role in the case of the substances in the studied leaf buds. The relatively low contents of specific phytocompounds, (such as e.g., AA) in leaf buds may however in combination with other reducing substances cause measurable biological effects.

The components of the leaf bud extracts of raspberry, blackberry, and raspberry-blackberry hybrid also exhibit strong antibacterial properties against *S. aureus* and *E. faecalis*, as well as weaker activity against *E. aerogenes*. Thus, these organs can be sources of substances inhibiting the growth of bacteria that are potentially pathogenic to humans, as an alternative to antibiotics. This is a promising prospect, as currently available antibiotics are not always effective due to increasing resistance of pathogenic bacteria to these drugs.

The analyzed leaf buds, due to their health-promoting properties (high content of minerals, including those with are deficient in the human diet, and of bioavailable antioxidant and antibacterial substances), could be used for the production of dietary supplements, chemotherapeutic agents or as an alternative to synthetic antioxidants, and preservatives used in the production of pharmaceuticals or cosmetics.

## Figures and Tables

**Table 1:** Mineral contents in buds of raspberry, blackberry and raspberry-blackberry hybrid.

Forms *	Mineral Content-mg 100 g^−1^ DW						
	K	Ca	Mg	Fe	Zn	Mn	Cu
1	609.04 ± 35.21a	219.97 ± 15.21a	91.58 ± 2.15a	23.45 ± 5.43a	2.03 ± 0.09a	0.39 ± 0.10a	0.10 ± 0.04a
2	630.32 ± 26.47a	222.40 ± 12.80a	95.63 ± 2.49a	24.02 ± 5.61a	1.83 ± 0.08b	0.58 ± 0.01b	0.09 ± 0.03a
3	638.04 ± 17.50a	188.74 ± 8.29b	89.16 ± 6.37a	15.90 ± 1.42b	2.76 ± 0.31c	0.72 ± 0.08c	0.12 ± 0.02a
4	687.54 ± 12.21c	154.48 ± 16.05c	91.20 ± 4.16a	11.77 ± 4.48c	1.85 ± 0.07b	0.46 ± 0.11ab	0.18 ± 0.03c
5	448.93 ± 14.28b	195.50 ± 9.32b	76.27 ± 3.37b	19.14 ± 1.20a	1.72 ± 0.11b	0.62 ± 0.02c	0.27 ± 0.05b
6	662.70 ± 19.34ac	206.73 ± 10.34ab	111.15 ± 4.55c	22.95 ± 4.34a	1.44 ± 0.12d	1.05 ± 0.12d	0.17 ± 0.02c

* 1—‘Cascade Delight’, 2—‘Glen Fyne’, 3—‘Octavia’, 4—‘Navaho’, 5—wild blackberry, 6—raspberry-blackberry hybrid. Different letters stand for different homogeneous groups. ‘Means sharing the same letter within a column are not significantly different at *p* < 0.05.

**Table 2:** Significant values of correlation coefficients at α = 0.05 level between TP, AA content and TAC marked with ABTS, DPPH.

Method of Analysis	AA	TP	ABTS	DPPH
AA	1.00			
TP		1.00		
ABTS		0.94	1.00	
DPPH	0.28	0.84	0.90	1.00

**Table 3:** Significant values of correlation coefficients at α = 0.05 between TP content and TAC (method of analysis: ABTS, DPPH) in W, EW and GW extracts *.

Table	Analysis	W			EW			GW		
		TP	ABTS	DPPH	TP	ABTS	DPPH	TP	ABTS	DPPH
W	TP	1.00	-	-	-	-	-	-	-	-
	ABTS	0.92	1.00	-	-	-	-	-	-	-
	DPPH	0.73	0.81	1.00	-	-	-	-	-	-
EW	TP	0.97	0.88	0.63	1.00	-	-	-	-	-
	ABTS	0.96	0.86	0.56	0.97	1.00	-	-	-	-
	DPPH	0.96	0.87	0.72	0.95	0.96	1.00	-	-	-
GW	TP	0.90	0.78	0.46	0.96	0.95	0.89	1.00	-	-
	ABTS	0.75	0.67	-	0.81	0.84	0.80	0.87	1.00	-
	DPPH	0.62	0.72	0.53	0.62	0.67	0.66	0.62	0.85	1.00

* Extracts: W—water, EW—ethanol-water, GW—glycerol-water.

**Table 4:** The biostatic effect of the extracts on selected strains of bacteria and amount of bioactive substances introduced with the extract.

Extract *	The Amount of Antioxidant Substances Per Disc			Microorganism/Zone of Inhibition [mm]					
	TPC [µg GA]	ABTS [µM trolox]	DPPH [µM trolox]	EA	EC	EF	SA	SE	SM
1W	0.19	0.05	0.24	0	0	4	3	0	0
1EW	0.35	0.09	0.44	2	0	8	5	0	0
1GW	0.47	0.12	0.60	0	0	14.5	8	0	0
2W	0.25	0.07	0.32	0	0	3	4.5	0	0
2EW	0.38	0.11	0.49	3.5	0	4	3	0	0
2GW	0.45	0.13	0.59	0	0	14	4	0	0
3W	0.34	0.11	0.45	0	0	2	4.7	0	0
3EW	0.51	0.12	0.63	1.5	0	6	4.5	0	0
3GW	0.52	0.12	0.64	0	0	10.5	5.5	0	0
4W	0.06	0.03	0.09	0	0	1	1.3	0	0
4EW	0.07	0.04	0.12	1.3	0	4	1.5	0	0
4GW	0.19	0.06	0.25	0	0	8	6	0	0
5W	0.10	0.05	0.16	0	0	2	2	0	0
5EW	0.15	0.06	0.21	2.4	0	1	2	0	0
5GW	0.28	0.12	0.40	0	0	9.5	7.4	0	0
6W	0.10	0.04	0.15	0	0	5	6	0	0
6EW	0.12	0.05	0.17	1.3	0	2	4	0	0
6GW	0.20	0.07	0.27	0	0	7.5	7.2	0	0
Kanamycin				23	23	26	23.8	27	26
Significant values of correlation coefficients at α = 0.05 between the TP amount and the growth inhibition zone				-	-	0.68	0.51	-	-
Significant values of correlation coefficients at α = 0.05 between the ABTS specific antioxidants amount and the growth inhibition zone				-	-	0.68	0.53	-	-
Significant values of correlation coefficients at α = 0.05 between the DPPH specific antioxidant amount and the growth inhibition zone				-	-	0.70	0.52	-	-

* Extracts: W—water, EW—ethanol-water, GW—glycerol-water, from the buds of 1—‘Cascade Delight’, 2—‘Glen Fyne’, 3—‘Octavia’, 4—‘Navaho’, 5—wild blackberry, 6—raspberry-blackberry hybrid. Microorganism: EA—*Enterobacter aerogenes*, EC—*Escherichia coli*, EF—*Enterococcus faecalis*, SA—*Staphylococcus aureus*, SE—*Salmonella enterica*, SM—*Serratia marcescens*.

## Data Availability

The data presented in this study are available on request from the corresponding authors.

## Sample Availability

Sample of the compounds are not available from the authors.
